# Prediction of Synthesis Yield of Polymethoxy Dibutyl Ether Under Small Sample Conditions

**DOI:** 10.3390/molecules30234601

**Published:** 2025-11-29

**Authors:** Xue Wang, Linyu Lu, Qiuxin Ma, Hongyan Shang, Lanyi Sun

**Affiliations:** 1 College of Chemical Engineering, China University of Petroleum, Qingdao 266580, China; 2 School of Chemical Engineering, Shandong Institute of Petrochemical and Chemical Technology, Dongying 257061, China

**Keywords:** polymethoxy dibutyl ether (BTPOM*_n_*), yield prediction, genetic algorithm (GA), CatBoost, small sample size, datadriven modeling

## Abstract

In chemical reaction processes, yield prediction frequently faces challenges, such as multi-variable coupling, significant nonlinearity, and the limited accuracy of traditional mechanistic models. This study develops a datadriven prediction model that integrates the genetic algorithm (GA) with CatBoost to address these challenges. Four variables, including reactant ratio (*n*-butanol to trioxane), reaction temperature, reaction time, and catalyst concentration, were selected as model inputs based on 88 sets of experimental data. The model outputs focused on the yield of polymethoxy dibutyl ether with a polymerization degree of 1 (BTPOM_1_) and the total yield of polymethoxy dibutyl ether with polymerization degrees of 1 to 8 (BTPOM_1–8_). The model achieved automatic optimization of CatBoost on hyperparameters by combining a hybrid-coding genetic algorithm. The results demonstrated that the GACatBoost model significantly outperformed GAAdaBoost for both datasets: for BTPOM_1_, it reduced the mean squared error (MSE) by 50.1%, mean absolute error (MAE) by 40.6%, and mean absolute percentage error (MAPE) by 17.8% relative to GAAdaBoost. For BTPOM_1–8_, the reductions were more pronounced, with MSE decreasing by 54.0%, MAE by 45.0%, and MAPE by 33.8% compared to GAAdaBoost. Additionally, the GACatBoost model significantly outperformed three classical machine learning algorithms: Support Vector Regression (SVR), Random Forest (RF), and KNearest Neighbor (KNN). Feature importance analysis revealed that reaction time and reaction temperature are the key factors influencing BTPOM*_n_* yield. This research provides a feasible approach for accurate synthesis yield prediction and process optimization under small sample conditions. It is particularly valuable for early-stage laboratory research where experimental data is often limited.

## 1. Introduction

Diesel engines continue to be indispensable in industrial and transportation systems due to their superior thermal efficiency, high torque output, and excellent fuel economy. These qualities make them particularly wellsuited for heavyduty applications such as freight transport, marine propulsion, and stationary power generation [1]. Nevertheless, conventional diesel fuels derived from petroleum resources have complex hydrocarbon compositions. These fuels release significant quantities of nitrogen oxides (NO_x_) and particulate matter (PM) during combustion. These pollutants pose severe risks to both public health and ecological systems [2]. NO_x_ contributes to the formation of photochemical smog and acid deposition, while PM triggers respiratory diseases and degrades air quality. Hence, mitigating these emissions is a critical challenge for sustainable energy development. Furthermore, diesel engines operating at high altitudes encounter exacerbated combustion inefficiencies due to reduced atmospheric oxygen levels. This leads to incomplete fuel oxidation, ignition failures, accelerated component wear, and overall performance deterioration [3].

Oxygenated fuels have been viewed as promising alternatives or blending components for diesel. Their inherent oxygen content enhances combustion completeness by supplying intramolecular oxygen. This capability effectively suppresses the formation of carbon monoxide (CO), unburned hydrocarbons (UHCs), and soot [4]. Among oxygenated fuels, polymethoxy dibutyl ether (BTPOM*_n_*)—with a molecular structure of C_4_H_9_O(CH_2_O)*_n_*C_4_H_9_, where *n* denotes the polymerization degree of methoxy groups—exhibits superior fuel properties due to its complete miscibility with hydrocarbons, high oxygen mass fraction, and favorable handling properties. Its advantages include a higher cetane number and an elevated net calorific value [5]. Additionally, BTPOM*_n_* has a density that closely matches that of commercial diesel fuels. This enhances its compatibility as a diesel blending component and solidifies its status as a promising oxygenated fuel candidate [6]. However, the synthesis of BTPOM*_n_* relies on a complex multi-step reaction network, encompassing processes such as trioxane depolymerization, formaldehyde oligomerization, and etherification with *n*-butanol. The yield of BTPOM*_n_* is highly sensitive to operating conditions, making accurate yield prediction critical for optimizing reaction parameters and reducing experimental costs. Despite this importance, yield prediction remains challenging due to the nonlinearity and coupling of process variables.

Traditional approaches to yield prediction primarily depend on mechanistic models, such as lumped kinetic models and molecular dynamics models [7]. These models often require an increased number of lumps in order to improve prediction accuracy. This would lead to more complex reaction networks, heavier computational loads, and slower operation speeds. Furthermore, mechanistic models are constrained by the current state of knowledge regarding reaction mechanisms, which hinders their ability to characterize how nonlinear relationships between system features influence yield variations. In contrast, datadriven models based on machine learning can establish relationships between input variables and output variables using historical data. It does not require explicit mechanistic interpretation. This advantage makes datadriven models more flexible for complex chemical processes, especially with the growing availability of real-time data.

In the field of petroleum catalysis, datadriven models have been widely applied for yield prediction. Dash et al. developed a hybrid neural model employing an artificial neural network model in conjunction with genetic algorithms for the prediction of water levels, and the results indicated that the model could effectively simulate waterlevel dynamics [8]. Sharifi et al. used a Support Vector Machine (SVM) to predict hydrocracking product yield [9], and Heilemann et al. adopted Lasso regression for crop yield prediction [10]. Ren et al. further proposed an oil production prediction model leveraging Adaptive Boosting (AdaBoost) [11]. Nevertheless, while neural network-based methods are plagued by drawbacks such as inadequate interpretability and prolonged training cycles, AdaBoost is limited by its strong susceptibility to outliers and intrinsic sequential learning paradigm, which renders parallel data processing infeasible [12].

CatBoost, an advanced variant of the gradient boosting algorithm, addresses these limitations by enabling automatic processing of categorical features and employing an ordered boosting mechanism. This design reduces overfitting and enhances the stability of the ensemble classifier [13,14,15]. However, the performance of CatBoost is highly dependent on the selection of globally optimal hyperparameters, including learning rate, tree depth, and number of iterations. Manual tuning or trial-and-error methods are not only labor-intensive but also likely to miss optimal parameter combinations. This becomes more pronounced under small sample conditions, where model generalization is already limited [16]. The genetic algorithm (GA), an evolutionary algorithm inspired by Darwin’s theory of natural selection, performs well in global optimization in complex spaces. It does not require the target function to be continuous or differentiable, making it wellsuited for tuning the hyperparameters of machine learning models [17].

Current research on BTPOM*_n_* synthesis has focused primarily on exploring reaction mechanisms and optimizing experimental conditions. Little attention has been paid to data-driven yield prediction. This gap is particularly evident under small sample conditions, which are common in early-stage laboratory research. This study proposes a GACatBoost hybrid model for BTPOM*_n_* yield prediction. A datadriven model containing four key operating variables—reactant ratio, reaction temperature, reaction time, and catalyst concentration—was developed to predict the yields of BTPOM_1_ and total BTPOM_1–8_. The hyperparameters of CatBoost using GA were optimized to enhance the model’s accuracy and generalization capabilities under small sample conditions. The performance of the GACatBoost model was then compared with that of the GAAdaBoost, SVR, RF, and KNN algorithms. The key factors influencing BTPOM*_n_* yields were identified through feature importance analysis, providing practical guidance for optimizing experimental processes.

## 2. Results and Discussion

### 2.1. Hyperparameter Optimization Results

Table 1 compares the hyperparameters of the GAAdaBoost model and the GACatBoost model, clearly showing the advantages of genetic algorithm-driven optimization. The GA made targeted adjustments to the key hyperparameters of both models, effectively enhancing their performance and efficiency. For the number of iterations, both models were set to 400, a configuration that reduced redundant computations during training. In terms of learning rate, the GA adapted different values for the two models: it set GAAdaBoost’s learning rate to 0.1 and GACatBoost’s to 0.3, allowing each model to update weights more efficiently per iteration and speed up training without compromising prediction accuracy. For tree depth, the GA also optimized it with differentiation: GAAdaBoost used a tree depth of 4, while GACatBoost used 5, and this setup helped reduce the risk of overfitting in small-sample scenarios, letting the models focus on generalizable patterns in data rather than local noise. Additionally, the GA configured GACatBoost with a maximum of 30 leaf nodes per tree, which further simplified the model’s complexity and helped improve its generalization ability for small datasets. These GAtuned hyperparameters helped both models strike a better balance between performance, training efficiency, and generalization.

### 2.2. Model Performance Evaluation

#### 2.2.1. Prediction Accuracy Comparison

**Figure 1:**
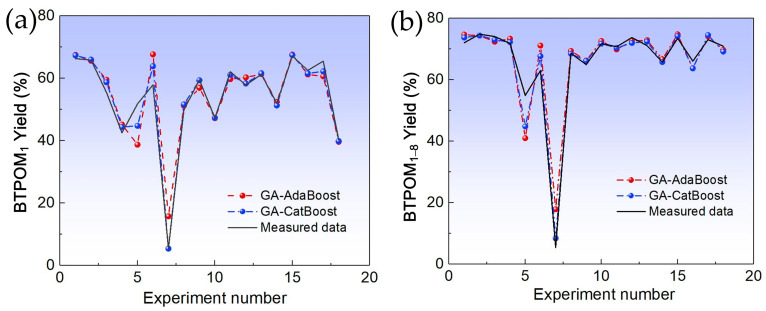
Comparison of prediction between GAAdaBoost and GACatBoost algorithms: (**a**) BTPOM_1_; (**b**) BTPOM_1–8_.

**Figure 2:**
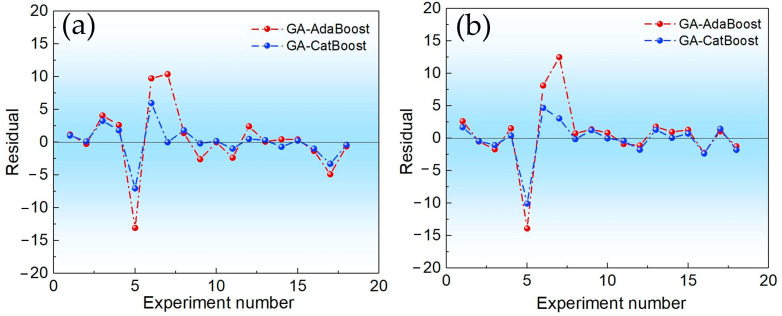
Comparison of residual between GAAdaBoost and GACatBoost algorithms: (**a**) BTPOM_1_; (**b**) BTPOM_1–8_.

The predictive performance of GACatBoost and GAAdaBoost was first evaluated through visual and quantitative analysis of yield predictions and residual distributions. Figure 1 contrasts the predicted vs. measured yields for BTPOM_1_ and BTPOM_1–8_ across both models. GACatBoost predictions (blue lines) closely tracked the measured data (black lines), whereas GAAdaBoost predictions (red lines) exhibited more pronounced deviations. This visual alignment was corroborated by the residual analysis in Figure 2. GACatBoost residuals were tightly clustered around zero, with a narrow range of −7 to 7%, while GAAdaBoost residuals spanned a wider range of −14 to 13% and showed more frequent large deviations. Narrower, zerocentered residuals indicate that GACatBoost’s prediction errors were more consistent and smaller in magnitude, reflecting greater reliability in real-world applications.

Table 2 provides quantitative validation of these trends, offering precise metrics to quantify GACatBoost’s improvements. GACatBoost reduced three core error metrics relative to GAAdaBoost for BTPOM_1_: the Mean Squared Error (MSE), which is sensitive to extreme errors, fell by 50.1%; the Mean Absolute Error (MAE), a robust measure of average deviation, decreased by 40.6%; and the Mean Absolute Percentage Error (MAPE), the relative error metric critical for practical yield assessment, dropped by 17.8%. These reductions signify GA–CatBoost’s ability to mitigate both systematic biases and extreme prediction outliers for BTPOM_1_.

The optimization effect was even more pronounced for BTPOM_1–8_. MSE reduction increased to 54.0%, which is nearly 8% higher than for BTPOM_1_. MAE reduction further rose to 45.0%, and MAPE reduction reached 33.8%, which is a notable improvement over BTPOM_1_. This enhanced performance underscores GACatBoost’s adaptability to complex data structures. The GAoptimized hyperparameters allowed the model to capture the intricate relationships between operating variables and total yield, which the GAAdaBoost tuning failed to fully leverage.

The coefficient of determination (R^2^), the measure of how well the model explains variance in the target variable, further validated GACatBoost’s superiority. R^2^ rose from 0.80 with the GAAdaBoost to 0.90 for BTPOM_1_. This indicates the model now explained ca. 10% more of the yield variability. R^2^ increased from 0.84 to 0.92 for BTPOM_1–8_, an 8% gain. These increases confirm that GACatBoost not only reduces errors but also establishes a stronger, more reliable relationship between input variables and yield. This is critical for guiding experimental optimization.

#### 2.2.2. Performance Comparison with Other Algorithms

The accuracy of these models was compared using three classical machine learning algorithms—Support Vector Regression (SVR), Random Forest (RF), and K-Nearest Neighbor (KNN)—to contextualize GA-CatBoost’s performance. All models used identical training/test splits and were tuned via appropriate methods with grid search for SVR, RF, KNN, and GA for CatBoost to ensure a fair comparison.

**Figure 3:**
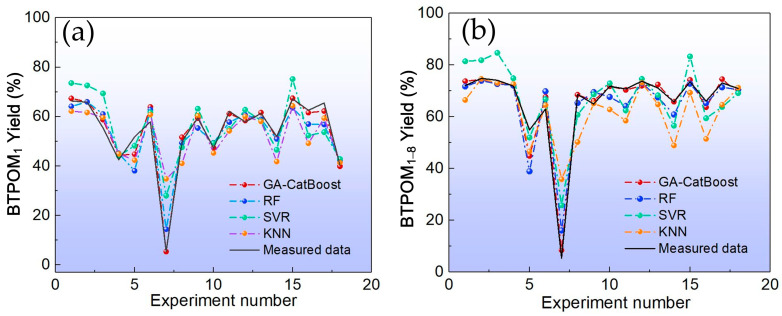
Comparison of predicted yields among different algorithms: (**a**) BTPOM_1_; (**b**) BTPOM_1–8_.

**Figure 4:**
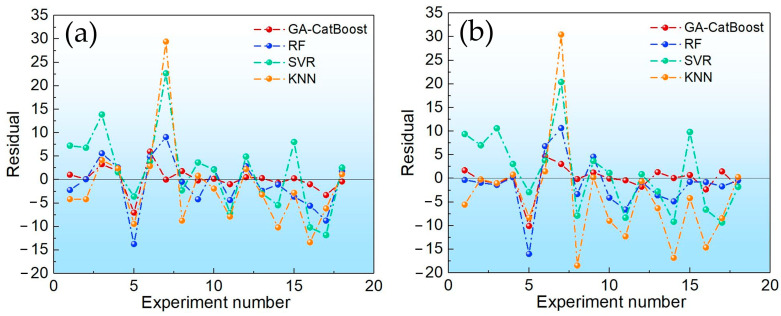
Comparison of prediction residuals among different algorithms: (**a**) BTPOM_1_; (**b**) BTPOM_1–8_.

Figure 3 and Figure 4 visualize these comparisons. GA-CatBoost consistently showed the closest alignment with measured yields and the smallest residuals across both BTPOM_1_ and BTPOM_1–8_. Quantitative results presented in Table 3 reinforced this trend. GA-CatBoost achieved the lowest MSE, MAE, and MAPE and the highest R^2^ for both targets. Compared to the worst-performing algorithm KNN, GA-CatBoost reduced the MSE of BTPOM_1_ by 75.5% from 83.84 to 20.52 and MAPE by 71.0% from 38.79% to 11.26%. The poor performance of KNN and SVR might stem from their sensitivity to hyperparameter tuning and difficulty capturing the nonlinear, coupled relationships in BTPOM*_n_* yield data. GA-CatBoost overcomes the limitations via its ensemble structure and optimized hyperparameters. GA-CatBoost maintained a clear advantage even against RF. It reduced the MSE by 41.1% from 30.70 to 18.07 and MAPE by 49.8% from 15.97% to 8.02% for BTPOM_1–8_. An ensemble of decision trees for RF provides some robustness to nonlinearity, but it lacks the ordered boosting mechanism of CatBoost.

#### 2.2.3. Computational Efficiency

GA-CatBoost outperformed all other models in accuracy. However, it required more training time than traditional CatBoost. Specifically, RF and SVR trained in ca. 10 s, whereas GA-CatBoost required ca. 120 s, including 100 GA iterations. This time difference arises from the iterative nature of GA. Each iteration involves training 50 separate CatBoost models to calculate fitness for each hyperparameter combination. This multiplies the computational load. However, this increased cost is justified for laboratory-scale BTPOM*_n_* synthesis optimization. Laboratory research prioritizes accurate yield predictions to reduce experimental costs and guide parameter refinement. Real-time performance is not a critical requirement here, as laboratory experiments typically run for hours to days, leaving ample time for model training. Future work could explore more efficient optimization algorithms to reduce training time without sacrificing hyperparameter optimization quality.

### 2.3. Feature Importance Analysis

**Figure 5:**
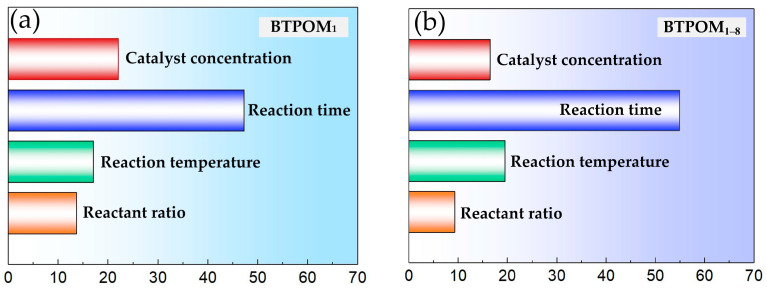
Feature importance analysis (F-score) for (**a**) BTPOM_1_ and (**b**) BTPOM_1–8_ yields.

The feature importance analysis shown in Figure 5 identified the key operating variables influencing BTPOM*_n_* yields. This provides the actionable insights for experimental optimization. Reaction time and reaction temperature emerged as the dominant factors, collectively accounting for about 80% of total feature importance. For BTPOM_1_, reaction time had an F-score of 47.2, and reaction temperature a score of 17.0. These scores were 54.9 and 19.4 for BTPOM_1–8_, respectively. In contrast, the reactant ratio with the F-score of 13.7 and 9.3 and catalyst concentration with the F-score of 22.1 and 16.4 had relatively minor effects.

The primacy of reaction time stems from the sequential nature of BTPOM*_n_* synthesis. Sufficient time is required for trioxane to depolymerize into formaldehyde and for formaldehyde to oligomerize and react with *n*-butanol. However, excessive reaction time triggers side reactions, such as the condensation of formaldehyde into polyoxymethylene glycols (MG*_n_*). It consumes reactive intermediates and reduces target yield. This balance can explain why reaction time is the most critical variable.

Reaction temperature, directly impacts catalyst activity and reaction kinetics. Temperatures below 90 °C slow trioxane depolymerization and formaldehyde oligomerization, leading to low yields. Temperatures above 130 °C accelerate NKC-9 catalyst deactivation and thermal decomposition of key intermediates. They can also reduce yield. The narrow optimal temperature range (100–120 °C) further highlights its importance.

The weak influence of catalyst concentration indicates that the NKC-9 catalyst reaches saturation at ca. 6 wt.%. Below this concentration, active sites are limited, and increasing catalyst load boosts yield; above 6 wt.%, no additional active sites are available, so higher concentrations do not improve performance. This saturation effect justifies the low F-score for catalyst concentration.

Generally, these findings guide practical experimental optimization. In order to maximize BTPOM*_n_* yield, reaction time should be controlled at 2–4 h to balance complete reaction and minimal side products. Temperature should be set at 100–120 °C to optimize catalyst activity. The reactant ratio should be at 2:1–1:1 to ensure sufficient formaldehyde for oligomerization. Catalyst concentration should be at 5–7 wt.% to avoid saturation and unnecessary cost.

## 3. Experiment

### 3.1. Chemical Reactions

The catalytic synthesis of BTPOM*_n_* from *n*-butanol and trioxane over NKC-9 molecular sieve also proceeds through a complex multi-step reaction network. Trioxane acts as a formaldehyde precursor, undergoing depolymerization to generate reactive oxymethylene intermediates. These key species drive subsequent BTPOM*_n_* formation. This study systematically elucidates the reaction mechanism and behavior governing BTPOM*_n_* synthesis. These reactions dictate the overall pathway of BTPOM*_n_* formation, and understanding their interplay is critical for optimizing yield.

Chromatographic monitoring was employed to track the time-dependent evolution of BTPOM*_n_* speciation following catalyst activation, providing direct insights into reaction progression. Oligomer populations emerged sequentially in correlation with reaction time. This reflected the stepwise nature of chain growth. In contrast, higher polymerization-degree homologues exhibited a monotonic concentration decay inversely proportional to their chain length, likely due to increased steric hindrance. It slows further etherification and promotes chain termination. The kinetic model developed herein formalizes this consecutive chain propagation mechanism, which is governed by a series of elementary reactions. Equations (1)–(3) indicate the reactions in details and describe the stepwise addition of oxymethylene units to *n*-butanol-derived intermediates [18,19]. The kinetic model employed a pseudo-homogeneous phase approximation, presuming uniform dispersion of catalyst active sites in the liquid phase with unimpeded reactant accessibility. As for BTPOM*_n_* chain propagation kinetics, the forward and reverse rate constants denoted as k_3_ and k_−3_ were assumed independent of polymer chain length due to structural and mechanistic congruence across oligomerization steps.

(1) $$TOX\overset{k_{1}}{\underset{k_{-1}}{⇄}}3FA$$

(2) $$2C_{4}H_{9}OH+FA\overset{k_{2}}{\underset{k_{-2}}{⇄}}BTPOM_{1}+H_{2}O$$

(3) $$BTPOM_{n}+FA\overset{k_{3}}{\underset{k_{-3}}{⇄}}BTPOM_{n+1}$$

The inherent water content in the reaction system introduced a competing catalytic pathway, wherein formaldehyde underwent condensation to form polyoxymethylene glycols (MG) as secondary products, as shown in Equation (4). This side reaction consumes reactive formaldehyde intermediates that would otherwise participate in BTPOM*_n_* synthesis, thereby reducing target product yield. Controlling water content or mitigating MG formation thus represents a potential strategy for improving BTPOM*_n_* production efficiency.

(4) $$H_{2}O+FA\overset{k_{MG}}{⇄}MG$$

In parallel, *n*-butanol underwent nucleophilic addition to formaldehyde, establishing the dominant pathway for generating polyoxymethylene hemiformal (HD*_n_*). This is a critical intermediate in BTPOM*_n_* synthesis. The governing reaction sequence for HD*_n_* generation is detailed in Equations (5) and (6), which describe the successive addition of formaldehyde to *n*-butanol to form HD_1_ and its subsequent conversion to HD_2_.

(5) $$C_{4}H_{9}OH+FA\overset{k_{4}}{\underset{k_{-4}}{⇄}}HD_{1}$$

(6) $$HD_{1}+FA\overset{k_{5}}{\underset{k_{-5}}{⇄}}HD_{2}$$

### 3.2. Materials

High-purity *n*-butanol (GC-grade, ≥98 mass%) and trioxane (GC-grade, ≥99 mass%) were procured from Shanghai Macklin Biochemical Technology Co., Ltd. (Shanghai, China). The GC-grade specification ensures minimal impurities, which is critical for avoiding unintended side reactions and ensuring accurate quantification of BTPOM*_n_* products via gas chromatography. The macroporous cation-exchange resin catalyst NKC-9 was commercially sourced from Nanjing Guojin New Materials Co., Ltd. (Nanjing, China). The macroporous structure provides a high specific surface area for catalytic active sites. Its cation-exchange properties facilitate the protonation of reactants. This is an essential step for initiating trioxane depolymerization and formaldehyde oligomerization.

Deionized water was generated in-house using a Millipore Milli-Q water purification system (IQ 7000, Thermo Fisher Scientific Inc., Waltham, MA, USA), ensuring ultra-low ion content to prevent interference with the catalytic reaction. Reference standards for BTPOM_1_ (C_4_H_9_-O-CH_2_O-C_4_H_9_) and BTPOM_2_ (C_4_H_9_-O-(CH_2_O)_2_-C_4_H_9_) were provided by the Systems Engineering Institute at the Academy of Military Sciences (Beijing, China). These standards served as critical calibrants for the quantitative chromatographic analysis of BTPOM*_n_* oligomers. This enables accurate determination of individual and total BTPOM*_n_* yields by establishing calibration curves for peak area-to-concentration conversion.

### 3.3. Apparatus and Experimental Procedure

The apparatus for BTPOM*_n_* synthesis was a 500 mL titanium alloy high-pressure autoclave (Model YZMR-4100D, Weihai Yantai Chemical Machinery Co., Ltd., Weihai, China). Titanium alloy was selected for the autoclave material due to its excellent corrosion resistance and its high thermal conductivity, which ensures uniform heat distribution during reaction. The autoclave was equipped with a dual-impeller mechanical stirrer featuring a 45° blade tilt angle and an impeller-to-reactor diameter ratio of 1:3. This stirrer design optimizes mixing efficiency, ensuring homogeneous contact between reactants and catalyst particles. The 45° blade tilt promotes axial and radial flow, preventing catalyst sedimentation and minimizing concentration gradients within the reaction mixture.

A nitrogen pressurization system equipped with a digital pressure regulator was used to create an inert atmosphere, preventing oxidative degradation of reactants or intermediates. A temperature control system incorporating a Pt100 resistance temperature detector (WZPT-035, Jiangsu Ming Cable Technology Co., Ltd., Taizhou, China) and a proportional-integral-derivative algorithm maintained temperature uniformity within ±0.5 °C. Precise temperature control is essential for BTPOM*_n_* synthesis, as reaction rates and catalyst activity are highly temperature-dependent.

**Figure 6:**
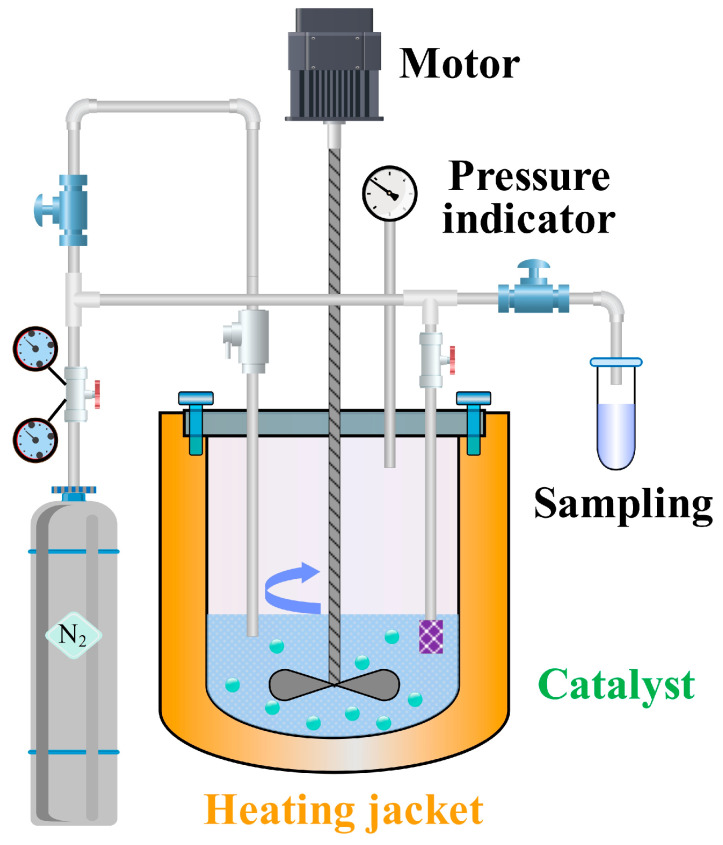
Schematic of the experimental setup for kinetic investigation.

A pressure stabilization unit consisting of a piezoelectric transducer and a fast-response solenoid valve limited pressure fluctuation to ±0.02 MPa. Stable pressure suppresses vaporization of low-boiling components, ensuring consistent reactant concentrations throughout the reaction. A sampling system featuring a 5 μm sintered metal filter retained catalyst particles during sampling, preventing contamination of collected samples. Nitrogen backfilling was employed post-sampling to maintain isobaric conditions, avoiding pressure-driven changes in reaction kinetics. A 2 kW heating jacket with a heating rate of 5 °C/min enabled controlled temperature ramps, reducing thermal shock to the reaction mixture and ensuring reproducible reaction initiation. Figure 6 presents the schematic of the experimental setup, illustrating the integration of these subsystems with the autoclave to enable precise control and monitoring of the BTPOM*_n_* synthesis process.

### 3.4. Experimental Procedure

The experimental procedure for BTPOM*_n_* synthesis was designed to ensure reproducibility and precise control of the four key input variables, including reactant ratio, reaction temperature, reaction time, and catalyst concentration. The *n*-butanol and formaldehyde were added to the autoclave at a molar ratio ranging from 1:2 to 2:1. The variable was selected to explore the impact of formaldehyde availability on BTPOM*_n_* chain length and yield. The stirrer was activated at 300 r/min to ensure homogeneous mixing of reactants, preventing localized concentration gradients that could skew reaction kinetics.

NKC-9 catalyst was added to the mixture at a concentration of 1 wt.% to 8 wt.%. The autoclave was hermetically sealed. The autoclave was then purged with high-purity nitrogen (99.99% purity) three times to remove residual oxygen. Oxygen would otherwise oxidize reactants or deactivate the catalyst and lead to reduced yield and inconsistent results. The autoclave was pressurized to 0.25 MPa with nitrogen to initiate the inert atmosphere following purging.

The heating jacket was activated to raise the reaction temperature from ambient to the target range, from 80 °C to 130 °C, at a constant rate of 5 °C/min. The stirrer speed was increased to 600 r/min to enhance mass transfer between reactants and catalyst once reaching the set temperature. This is critical for accelerating reaction rates while maintaining homogeneity. The pressure was simultaneously adjusted to 1.0–1.1 MPa to suppress vaporization of low-boiling components, ensuring that reactants remained in the liquid phase and available for reaction. Reaction time was recorded from this point, with a variable range of 1 h to 6 h to capture the full progression of BTPOM*_n_* formation and potential yield decline due to side reactions.

Samples of 5 mL were collected at 5 min intervals after the first hour and at 30 min intervals for the rest time using the autoclave’s sampling valve to track yield evolution over time. Collected samples were filtered through a 0.22 μm organic phase filter to remove any remaining catalyst fines, preventing interference with chromatographic analysis. Filtrates were then analyzed via gas chromatography (Model 7890A, Agilent Technologies, Santa Clara, CA, USA) equipped with a DB-WAX capillary column (30 m × 0.25 mm × 0.25 μm) and a flame ionization detector (FID)—a configuration optimized for separating and quantifying oxygenated organic compounds like BTPOM*_n_*. The GC operating conditions were carefully calibrated. Inlet temperature was set to 250 °C to ensure complete vaporization of samples, and detector temperature was set at 280 °C for maximum sensitivity. A column temperature program with an initial temperature of 60 °C held for 2 min, then ramped at 10 °C/min to 220 °C and held for 5 min was employed to achieve baseline separation of the BTPOM_1_ to BTPOM_8_. Nitrogen was used as the carrier gas at a flow rate of 1.0 mL/min with a split ratio of 10:1, balancing sensitivity and peak resolution.

### 3.5. Yield Calculation

The yield of BTPOM_1_ and the total yield of BTPOM_1–8_ were calculated using the internal standard method, with n-hexadecane selected as the internal standard. This method was chosen for its robustness against variations in sample injection volume and chromatographic conditions, ensuring accurate and reproducible yield quantification. The calculation is expressed in Equation (7):

(7) $$Y_{i}=\frac{m_{i,prod}}{m_{i,theo}}\times 100\%$$

Here, *Y_i_* represents the yield of either BTPOM_1_ or BTPOM_1–8_. *m_i_*_,*prod*_ denotes the actual mass of BTPOM_1_ or BTPOM_1–8_ in the product. It was determined via GC analysis by comparing the peak area of the target BTPOM*_n_* species to that of the internal standard using pre-established calibration curves [20]. *m_i_*_,*theo*_ is the theoretical mass of BTPOM_1_ or BTPOM_1–8_. It was calculated based on the initial amount of trioxane. Trioxane is the sole source of formaldehyde; its initial mass dictates the maximum possible yield of BTPOM*_n_* species. This theoretical mass calculation accounts for the stoichiometry of trioxane depolymerization and the subsequent etherification with *n*-butanol, ensuring a direct link between reactant input and expected product output.

## 4. Methodology

A data-driven modeling framework was established to address the challenge of BTPOM_1_ and BTPOM_1–8_ yield prediction under small sample conditions with 88 experimental sets. This framework defines a mapping relationship between input features and output yields. The input feature vector involves reactant ratio, reaction temperature, reaction time, and catalyst concentration. The output consists of BTPOM_1_ yield and total BTPOM_1–8_ yield. The methodology focuses on integrating the CatBoost algorithm with the genetic algorithm (GA) for hyperparameter optimization to address the limitations of manual tuning and traditional models.

### 4.1. CatBoost Algorithm

CatBoost is an advanced variant of the Gradient Boosting Decision Tree (GBDT) algorithm, specifically designed to overcome the reliance on manual categorical feature encoding and high susceptibility to overfitting [21]. Target encoding of categorical features and an ordered boosting mechanism were applied to enhance model stability and prediction accuracy for complex chemical process data.

(1) Target Encoding of Categorical Features.

CatBoost employs target encoding that maps categorical values to numerical representations using the target variable’s statistical properties for categorical features. The target encoding of category *c_m_* is calculated via Equation (8) for a categorical feature C with distinct values {*c*_1_, *c*_2_, *…*, *c_k_*}:

(8) $$\emptyset \left(c_{m}\right)=\frac{\sum_{x_{j}:C\left(x_{j}\right)=c_{m}}y_{j}+\alpha \cdot \mu }{count\left(c_{m}\right)+\alpha }$$

Here, ∅(*c_m_*) is the encoded value of category *c_m_*. *α* is a smoothing coefficient that balances category-specific statistics from the sum of target values *y_j_* for samples in *c_m_* and the global mean of the target variable *μ*. This balance is critical for small sample conditions, as it prevents bias from categories with few samples. The term count (*c_m_*) represents the number of samples in category *c_m_*. One-hot encoding expands categorical features into high-dimensional binary vectors and risks overfitting with limited data. In contrast, target encoding reduces dimensionality while preserving the statistical relevance of categorical features, making it far more suitable for the small sample size here.

(2) Objective Function

The objective function of CatBoost in the *t*-*th* iteration incorporates a loss term and two regularization terms. They work together to minimize prediction error and suppress overfitting. It is defined in Equation (9):

(9) $$L_{t}=\sum_{i=1}^{n}L\left(y_{i},F_{t-1}\left(x_{i}\right)+h_{t}\left(x_{i}\right)\right)+\lambda \cdot \sum_{j=1}^{N_{leaf}}v_{j}^{2}+\gamma \cdot N_{leaf}$$

In this equation, *L*(·) is the loss function. It was set to mean squared error (MSE) for regression tasks to penalize large prediction deviations. *F_t−_*_1_(*x_i_*) is the predicted yield of sample *x_i_* using the first *t* − 1 decision trees, and *h_t_*(*x_i_*) is the output of the *t-th* decision tree for *x_i_*. λ is the regularization coefficient for leaf node values (*v_j_*). It smooths these values to prevent extreme predictions that contribute to overfitting. *γ* is the regularization coefficient for the number of leaf nodes (*N_leaf_*) in the *t-th* tree. It penalizes excessive leaf nodes to control tree complexity and avoid overfitting to noise in small samples.

### 4.2. Genetic Algorithm (GA)

The genetic algorithm (GA) is an evolutionary optimization technique inspired by natural selection and genetic variation. It shines in global optimization for complex, non-differentiable solution spaces. It is ideal for tuning the hyperparameters of CatBoost, which lack a clear mathematical relationship to model performance [22,23,24]. The workflow of GA consists of population initialization, fitness calculation, and three genetic operations, including selection, crossover, and mutation. All these are designed to iteratively refine solutions toward the global optimum.

#### 4.2.1. Key Parameters of GA

In the genetic algorithm adopted in this study, the settings of key parameters were determined to balance algorithm performance and practical application requirements. The population size was set to 50 to achieve a balance between computational efficiency and search diversity. The iteration number was specified as 100, and the algorithm terminated when the fitness converged. The crossover probability (P_c_) was set to 0.8, which was sufficiently high to effectively promote gene recombination. The mutation probability (P_m_) was set to 0.01. It was low enough to avoid excessive randomness interference while being high enough to help the algorithm escape local optima. The encoding method adopted real-number encoding, which can directly map hyperparameters to GA individuals and thus avoid errors that may be introduced by binary encoding.

#### 4.2.2. Genetic Operations

Roulette wheel selection was used in selection. The probability of an individual being selected for reproduction is proportional to its fitness. The selection probability (*P_sel_*(*x_i_*)) for individual *x_i_* is defined in Equation (10):

(10) $$P_{sel}\left(x_{i}\right)=\frac{f\left(x_{i}\right)}{\sum_{j=1}^{N}f\left(x_{j}\right)}$$

Here, *f*(*x_i_*) is the fitness of individual *x_i_*, and *N* is the population size. This method prioritizes individuals with better fitness to ensure that favorable hyperparameter combinations are passed to subsequent generations.

Single-point crossover was implemented. A random crossover point k was selected for two parent individuals *x*_1_ = [a_1_, a_2_, …, a_k_, …, a_l_] and *x*_2_ = [b_1_, b_2_, …, b_k_, …, b_l_]. L is the encoding length, equal to the number of CatBoost hyperparameters. Two offspring *x*_1_′ = [a_1_, …, a_k_, b_k+1_, …, b_l_] and *x*_2_′ = [b_1_, …, b_k_, a_k+1_, …, a_l_] were generated by swapping segments of the parents. This operation efficiently combined beneficial traits from both parents to explore new hyperparameter combinations.

Mutation involved introducing small random perturbations to individual hyperparameter values for real-number encoding. The mutation probability (P_m_ = 0.01) ensured that changes were infrequent enough to preserve good solutions but frequent enough to avoid stagnation in local optima.

The GA operated in a cycle by initializing a population of hyperparameter combinations, calculating the fitness of each individual, and applying selection/crossover/mutation to generate a new population. It repeated until the maximum number of iterations was reached or fitness converged. This cycle ensured that the algorithm efficiently searched for the global optimum in CatBoost’s hyperparameter space.

### 4.3. GA-CatBoost Hybrid Model

The GA-CatBoost hybrid model addresses the inefficiency of manual CatBoost hyperparameter tuning by leveraging GA’s global optimization capabilities. The workflow illustrated in Figure 2 integrates data preprocessing, GA-driven hyperparameter optimization, and CatBoost model training/evaluation to ensure accurate yield prediction under small samples.

#### 4.3.1. Hyperparameter Optimization Scope

The key CatBoost hyperparameters and their search ranges shown in Table 4 were determined via literature review and preliminary experiments to ensure coverage of values that balance model accuracy and complexity [14,25].

The learning rate range [0.01, 0.3] prevents excessively slow training or unstable convergence, while the tree depth range [3, 10] avoids underfitting and overfitting in small samples.

#### 4.3.2. Fitness Function

The fitness function in this genetic algorithm optimization framework is designed as a multi-objective evaluation criterion that balances prediction accuracy against model complexity. The function is defined in Equation (11):

(11) $$f=-{MAE}_{mean}-\lambda \left(N_{total}-N_{selected}\right)$$

where *MAE_mean_* represents the mean absolute error averaged across both output targets through 5-fold cross-validation, *N_total_* denotes the total available features and is 4 in this study. *N_selected_* indicates the number of features actively used. *λ* serves as a regularization coefficient and is set to 0.01. This formulation addresses two competing objectives simultaneously: the primary component (−*MAE_mean_*) drives the optimization toward higher prediction accuracy by minimizing the cross-validated error across both output targets, while the secondary penalty term (−0.01 × (4 − *N_selected_*)) encourages feature sparsity and model simplicity by penalizing unused features.

#### 4.3.3. Implementation Steps

**Figure 7:**
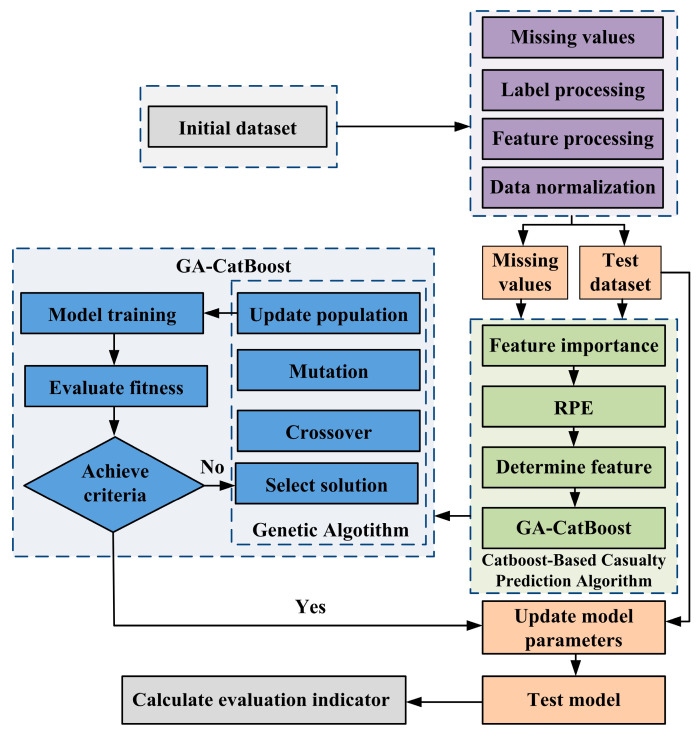
Flowchart of the GA-CatBoost model for BTPOM*_n_* yield prediction.

First, data preprocessing was conducted. The 88 experimental samples were split into a training set of 70 samples (accounting for 80%) and a test set of 18 samples (representing 20%) using stratified sampling. A GA population consisting of 50 individuals was randomly generated within the hyperparameter search range for GA initialization. Each individual was used to train a CatBoost model with the training set for fitness calculation. The model’s mean squared error (MSE) on the validation set was calculated to determine the individual’s fitness. Subsequently, the genetic operations, including selection, crossover, and mutation were performed to generate a new population. These two steps with fitness calculation and genetic operations were repeated for 100 iterations. The individual with the highest fitness was selected as the optimal hyperparameter combination Θ* after the iterations. Finally, a CatBoost model shown in Figure 7 was trained using the optimal hyperparameter combination Θ* and the training set. Its performance was evaluated on the test set.

### 4.4. Implementation Details

All codes for the GA-CatBoost model and comparative algorithm simulations were developed in-house using Python 3.9, with the programming environment built on Anaconda 2022.10 (64-bit, Windows 10) to ensure consistent package management. Core open-source libraries and their versions include catboost 1.1.1 for CatBoost model implementation and categorical feature processing; scikit-learn 1.2.2 for data preprocessing, cross-validation, performance metric calculation, and comparative algorithm implementation with grid search tuning; numpy 1.24.3 and pandas 1.5.3 for data loading, cleaning and numerical operations; and deap 1.3.3 for custom genetic algorithm design for CatBoost hyperparameter optimization. All codes follow standard Python practices, with detailed documentation for key steps to facilitate reproducibility.

## 5. Conclusions

This study addressed the challenge of BTPOM*_n_* yield prediction under small sample conditions with 88 experimental sets by developing a GA-CatBoost hybrid model. The main conclusions are the following. The GA-CatBoost model effectively predicts BTPOM_1_ and BTPOM_1–8_ yields using four input variables, including reactant ratio, reaction temperature, reaction time, and catalyst concentration. The model overcomes the limitations of traditional mechanistic models and manually tuned machine learning models by integrating GA-driven hyperparameter optimization with CatBoost’s robust ensemble structure. GA optimization significantly enhanced CatBoost’s performance. Relative to the GA-AdaBoost model, GA-CatBoost reduced MSE by 50.1 to 54.0%, MAE by 40.6 to 45.0%, and MAPE by 17.8 to 33.8%. Meanwhile, the coefficient of determination increased by 10% (from 0.80 to 0.90) for BTPOM_1_ and 8% (from 0.84 to 0.92) for BTPOM_1–8_, confirming a stronger input–output relationship. It exhibits larger improvements for the more complex BTPOM_1–8_ target. It also outperformed SVR, RF, and KNN, confirming its superiority in accuracy and generalization for small sample data. Feature importance analysis identified the reaction time with an F-score of about 54.9 and reaction temperature with an F-score of about 19.4 as the dominant factors influencing BTPOM*_n_* yields. However, catalyst concentration with the F-score of about 6 had minimal impact. Based on these insights, the following optimal operating conditions are recommended: 2–4 h reaction time, 100–120 °C reaction temperature, 2:1–1:1 reactant ratio, and 5–7 wt.% catalyst concentration.

Future work should extend this research by expanding the sample size to 200–300 sets. Additionally, more efficient optimization algorithms, such as PSO and gray wolf optimizer, can be explored to reduce training time. The experimental parameter range in subsequent research should be extended to further enhance the generalizability and practical applicability of the GA-CatBoost model, thereby broadening the model’s yield prediction scope to cover more diverse scenarios from low to high yields. This expansion will enable the model to provide more comprehensive guidance for the optimization of BTPOM*_n_* synthesis processes under different operating conditions. Additional variables, including stirring speed and reaction pressure, can be incorporated to build a more comprehensive prediction model. This would further enhance the model’s utility for BTPOM*_n_* synthesis optimization and broader oxygenated fuel research.

## Figures and Tables

**Table 1:** Hyperparameters of GAAdaBoost model and GACatBoost model.

Algorithm	Hyperparameters
GAAdaBoost	Learning rate: 0.1; Tree depth: 4; Number of iterations: 400; Other parameters are default
GACatBoost	Learning rate: 0.3; Tree depth: 5; Number of iterations: 400; Maximum leaf nodes: 30

**Table 2:** Comparison of prediction results of GAAdaBoost and GACatBoost.

Algorithm		MSE		MAE		MAPE		R^2^	
		Training	Testing	Training	Testing	Training	Testing	Training	Testing
GAAdaBoost	BTPOM_1_	17.45	41.10	3.40	5.13	8.85	13.70	0.92	0.80
	BTPOM_1–8_	17.90	39.26	3.41	4.84	8.10	12.11	0.94	0.84
GACatBoost	BTPOM_1_	0.97	20.52	0.44	3.05	1.14	11.26	0.99	0.90
	BTPOM_1–8_	2.31	18.07	0.55	2.66	1.04	8.02	0.99	0.92

**Table 3:** Comparison of prediction results of various algorithms.

Algorithm	BTPOM_1_				BTPOM_1–8_			
	MSE	MAE	MAPE(%)	R^2^	MSE	MAE	MAPE(%)	R^2^
GA-CatBoost	20.52	3.05	11.26	0.90	18.07	2.66	8.02	0.92
RF	28.78	4.19	15.88	0.86	30.70	3.77	15.97	0.87
SVR	71.40	6.70	32.65	0.64	64.93	6.59	28.89	0.73
KNN	83.84	6.39	38.79	0.58	123.93	7.73	40.37	0.48

**Table 4:** Hyperparameters and their search ranges.

Hyperparameter	Description	Search Range
Learning rate	Step size of gradient update	[0.01, 0.3]
Tree depth	Maximum depth of each decision tree	[3, 10]
Number of iterations	Number of decision trees (ensemble size)	[100, 2000]
Maximum leaf nodes (N_leaf_)	Maximum number of leaf nodes per tree	[10, 100]

## Data Availability

Data are contained within this article.
